# Comparative Analysis of TLS and UAV Sensors for Estimation of Grapevine Geometric Parameters

**DOI:** 10.3390/s24165183

**Published:** 2024-08-11

**Authors:** Leilson Ferreira, Joaquim J. Sousa, José. M. Lourenço, Emanuel Peres, Raul Morais, Luís Pádua

**Affiliations:** 1 Department of Agronomy, School of Agrarian and Veterinary Sciences, University of Trás-os-Montes e Alto Douro, 5000-801 Vila Real, Portugal; leilsonferreiragomes@utad.pt; 2 Centre for the Research and Technology of Agro-Environmental and Biological Sciences, University of Trás-os-Montes e Alto Douro, 5000-801 Vila Real, Portugal; eperes@utad.pt (E.P.); rmorais@utad.pt (R.M.); 3 Engineering Department, School of Science and Technology, University of Trás-os-Montes e Alto Douro, 5000-801 Vila Real, Portugal; jjsousa@utad.pt; 4 Centre for Robotics in Industry and Intelligent Systems (CRIIS), Institute for Systems and Computer Engineering, Technology and Science (INESC-TEC), 4200-465 Porto, Portugal; 5 Geology Department and Geosciences Center (CGeo), University of Trás-os-Montes e Alto Douro, 5000-801 Vila Real, Portugal; martinho@utad.pt; 6 Institute for Innovation, Capacity Building and Sustainability of Agri-Food Production, University of Trás-os-Montes e Alto Douro, 5000-801 Vila Real, Portugal

**Keywords:** precision viticulture, point clouds, unmanned aerial vehicles, terrestrial laser scanner, multispectral data, panchromatic data, LiDAR, thermal infrared, grapevine height, grapevine volume

## Abstract

Understanding geometric and biophysical characteristics is essential for determining grapevine vigor and improving input management and automation in viticulture. This study compares point cloud data obtained from a Terrestrial Laser Scanner (TLS) and various UAV sensors including multispectral, panchromatic, Thermal Infrared (TIR), RGB, and LiDAR data, to estimate geometric parameters of grapevines. Descriptive statistics, linear correlations, significance using the F-test of overall significance, and box plots were used for analysis. The results indicate that 3D point clouds from these sensors can accurately estimate maximum grapevine height, projected area, and volume, though with varying degrees of accuracy. The TLS data showed the highest correlation with grapevine height (*r* = 0.95, *p* < 0.001; *R*^2^ = 0.90; RMSE = 0.027 m), while point cloud data from panchromatic, RGB, and multispectral sensors also performed well, closely matching TLS and measured values (*r* > 0.83, *p* < 0.001; *R*^2^ > 0.70; RMSE < 0.084 m). In contrast, TIR point cloud data performed poorly in estimating grapevine height (*r* = 0.76, *p* < 0.001; *R*^2^ = 0.58; RMSE = 0.147 m) and projected area (*r* = 0.82, *p* < 0.001; *R*^2^ = 0.66; RMSE = 0.165 m). The greater variability observed in projected area and volume from UAV sensors is related to the low point density associated with spatial resolution. These findings are valuable for both researchers and winegrowers, as they support the optimization of TLS and UAV sensors for precision viticulture, providing a basis for further research and helping farmers select appropriate technologies for crop monitoring.

## 1. Introduction

Precision agriculture (PA) is an agricultural management strategy that relies on data observation, acquisition, and processing to understand and respond to temporal and spatial variability. The goal is to enhance the sustainability of agricultural production by optimizing resource use, reducing risks, and minimizing environmental impacts [1]. This approach is crucial for the efficient management of agricultural systems, including viticulture [2]. Viticulture involves the cultivation of grapes for wine production, a complex process that relies on a detailed understanding of the vineyard environment [3]. Traditionally, this understanding has been obtained through labor-intensive and often imprecise methods such as direct observations and soil sampling [4]. However, with the advancement of PA, various technologies for precision viticulture (PV) have become increasingly important [5,6,7].

One of the initial steps in adopting PV involves measuring the geometric and biophysical parameters of grapevines (*vitis vinifera* L.) [6]. The data collected are then interpreted and evaluated from a viticulture perspective, aiding winegrowers in the decision-making process, to enhance crop production and quality [8]. The precise characterization of these parameters is essential for efficient and sustainable management, optimizing resource use, and increasing productivity [9]. Studies have indicated that the volume and external canopy structure are important factors in understanding plant growth and biological characteristics, as these can indicate vigor [10]. The size of grapevine canopies is closely correlated with the amount of intercepted sunlight, the assimilated carbon [11,12,13], and biomass, which is important for planning pesticide application and defoliation strategies [14]. Additionally, canopy volume is directly linked to water evaporation [15], making these parameters essential for assessing crop management, plant health, water use efficiency, nutritional needs, and optimizing yield [10,15]. Obtaining reliable and timely data on these geometric parameters is, therefore, of utmost importance.

Traditional methods for acquiring geometrical data on plants, such as manual measurements and visual inspections, are time-consuming and labor-intensive [16]. However, technological advancements have led to the development of sensors capable of rapid three-dimensional (3D) data capture, enabling accurate measurements of plant structures. These include Terrestrial Laser Scanners (TLS) [7,17,18,19,20], as well as Light Detection And Ranging (LiDAR) and optical sensors mounted on Unmanned Aerial Vehicles (UAVs) [21,22,23,24,25]. Data collected using these technologies can generate point clouds via Structure from Motion (SfM) techniques or calculate point clouds directly using LiDAR, representing the field in a 3D space (X, Y, Z) [26,27,28,29]. TLS instruments allow for the non-destructive and precise digitization of physical scenes into 3D point clouds and have been extensively studied in precision farming applications [30]. TLS systems use LiDAR sensors that emit light pulses, which reflect off plants, allowing the calculation of distances based on the time taken for the sensor pulses to return to the sensor [31]. While TLS methods are widely used in civil and industrial engineering, such as in scanning buildings and archaeological sites [26,32,33], they also offer significant potential in agriculture for mapping, monitoring crop growth, and optimizing management practices [34,35].

Several studies have explored the use of stationary and mobile TLS point clouds in PA and PV. These applications include geometric analysis [36,37,38], biomass estimation [17], and fruit detection [39]. In PV, most studies have focused on using 3D point clouds from mobile TLS to extract structural parameters and biophysical variables [7,34,40,41,42,43,44,45,46], with fewer studies using stationary TLS [47,48].

Pagliai et al. [34] conducted a comparative study using UAVs, mobile TLS, and a mobile application [49], to assess grapevine canopy parameters. Three data acquisition campaigns were conducted on geo-referenced grapevines, using the Leaf Area Index (LAI) as the reference value. The study found that all tools accurately distinguished zones with varying canopy sizes. The correlation between height data was highest with the mobile application, followed by TLS and UAV canopy volume measurements. The correlation between LAI and volume was moderately strong for all tools, with the highest correlation observed for mobile TLS and the lowest for UAV data. Torres-Sánchez et al. [31] used UAV imagery and mobile TLS to estimate geometric parameters, including maximum height, projected area, and volume (2.5D and convex hull) in vineyards and peach and pear orchards. A high correlation was observed between the sensors, with geometric parameters estimated by mobile TLS generally higher than those of the UAV imagery. The smallest difference between the two sensors was found in maximum height estimates. Despite these satisfactory results, these studies have only compared sensor measurements without validation against actual field measurements. Llorens et al. [50] compared mobile TLS, ultrasonic sensors, and manual canopy measurements and found that grapevine canopy width and volume measurements based on TLS data had lower coefficients of determination (*R*^2^) than those obtained with the ultrasonic and manual approaches. Rinaldi et al. [48] developed a protocol to characterize grapevine canopy geometry and determine BBCH stages using manually measured data and a stationary TLS and found a significant correlation between TLS data and LAI at each growth stage. Moreover, they found a statistically significant relationship between row volume, leaf wall area, and the grapevine’s growth stage, with high correlations for height and width between manual measurements and TLS data.

Sensors coupled to UAVs have also been used to extract geometric parameters of grapevines and other biophysical variables [8,51,52,53]. These sensors, employing computer vision and photogrammetric techniques, generate point clouds [54]. Several studies have shown the potential of 3D canopy reconstruction for assessing vineyard spatial variability [55,56] and temporal vegetative decline, detecting grapevine trunks and vineyards [57,58,59], monitoring canopy management operations [60], estimating yield [61], pruning wood weight [62], measuring leaf area index [63,64], and optimizing treatments with pesticides and fertilizers [65]. Cantürk et al. [66] demonstrated an approach using point cloud data to detect trunk positions and extract the total height, width, and volume of the grapevine canopies. The combination of UAV imagery with 3D point cloud data is widely used to assess canopy height, allowing for a feasible analysis of biophysical parameter extraction in agricultural contexts [16,36].

According to Torres-Sánchez et al. [31], TLS and UAV systems differ significantly in terms of sensor types used and captured perspectives. Most UAV-based studies in PV use passive sensors, providing an aerial view, whereas TLS systems use active sensors, offering a ground perspective. Previous studies comparing these systems found divergences in the extracted geometric parameters of plants [34,67,68], highlighting the effectiveness of TLS and UAV data for both qualitative and quantitative analysis of plant structures. However, there is a gap in the literature concerning the comparison of static TLS systems and various UAV sensors for accurately estimating grapevine geometric parameters, particularly with field measurement validation.

This article addresses this gap by comparing a static TLS scanner and multiple UAV sensors, including RGB, LiDAR, Thermal Infrared (TIR), multispectral, and panchromatic data, to evaluate their capabilities in estimating geometric parameters such as the height, canopy area, and volume of grapevines. The study validates maximum height estimates with field measurements and canopy projected area measured in a Geographical Information System (GIS). Additionally, it discusses the strengths and limitations of each evaluated sensor in estimating these metrics, providing valuable insights for both researchers and winegrowers.

## 2. Materials and Methods

### 2.1. Study Area

**Figure 1:**
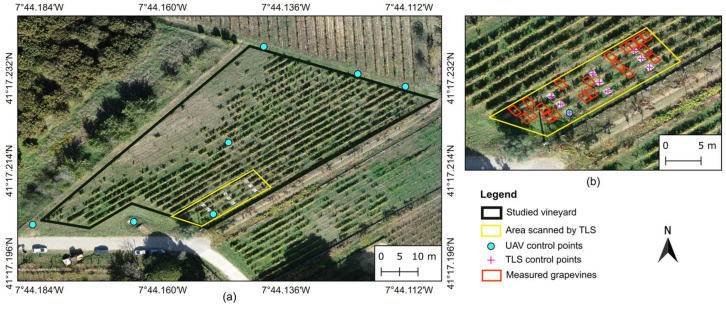
Overview of studied vineyard plot subjected to unmanned aerial vehicle data acquisition (**a**) and the area scanned by terrestrial laser scanner along with the studied grapevines (**b**).

This study was conducted in a 0.30-hectare experimental vineyard located on the campus of the University of Trás-os-Montes e Alto Douro, Vila Real, Portugal (41°17′13.2″ N 7°44′08.7″ W WGS84, altitude: 462 m). The vineyard (Figure 1), planted with Malvasia Fina variety, is trained in a double Guyot system. The grapevines are spaced approximately 1.20 m apart within each row, with 1.80 m between the 22 rows, which are oriented NE–SW. This rainfed vineyard is managed with foliar fertilization and phytosanitary treatments throughout the growing season. The inter-row areas contain spontaneous vegetation, which is managed mechanically at least twice per season.

### 2.2. Data Acquisition

**Figure 2:**
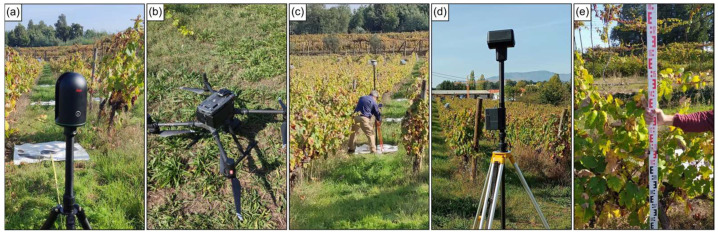
Equipment used and data acquisition tasks: (**a**) BLK360 G1; (**b**) Matrice 300 RTK; (**c**) acquisition of coordinates from deployed targets; (**d**) D-RTK 2 high precision GNSS mobile station; and (**e**) measurement of grapevine height.

Both UAV surveys and TLS scans were conducted on 10 October 2023, after the harvesting period (BBCH 91) [69]. Fifteen targets, each measuring 1 × 1 m, were placed throughout the vineyard plot to assist with UAV data alignment and TLS scans georeferencing. These targets were coordinated using a real-time kinematic (RTK) Global Navigation Satellite System (GNSS) receiver (Trimble R2, Trimble Inc., Westminster, CO, USA) in the EPSG:3763 (ETRS89/Portugal TM06) coordinate system, as shown in Figure 2c. Seven of these targets were designated as ground control points (GCPs) for UAV data alignment [70]. They were positioned in three groups: three placed in the northern part of the vineyard, three in the southern part, and one near the central area (Figure 1a). The remaining eight targets, along with one of the GCPs, were used for georeferencing and merging the TLS scans (Figure 1b). After completing data acquisition from all sensors, the heights of 20 grapevines within the area surveyed by both UAV and TLS (Figure 1b) were measured (Figure 2e). At same time, the coordinates of each grapevine were acquired using an RTK tablet (LT700 RTK, Shanghai Huace Navigation Technology Ltd., Shanghai, China).

#### 2.2.1. UAV Data Acquisition

UAV data were obtained using the Matrice 300 RTK (DJI, Shenzhen, China), shown in Figure 2b. This UAV supports multiple payload sensors. In this study, the Zenmuse H20T was used to acquire both TIR (640 × 512 pixels, 12,000 nm) and RGB (4056 × 3040 pixels) imagery, Zenmuse L1 was used to acquire LiDAR point cloud data with a maximum of three returns and RGB imagery (5472 × 3648 pixels), and the Micasense ALTUM-PT (AgEagle Aerial Systems Inc., Wichita, KS, USA) was used to acquire multispectral imagery (2064 × 1544 pixels) in blue (475 nm ± 32 nm), green (560 nm ± 27 nm), red (668 nm ± 14 nm), red-edge (717 nm ± 14 nm) and near-infrared (NIR, 842 nm ± 57 nm) bands, and also panchromatic (4112 × 3008 pixels, 634 nm ± 463 nm) and TIR (320 × 256 pixels, 11,000 nm ± 6000 nm) imagery. Zenmuse sensors are integrated into a three-axis gimbal for stabilization. The UAV RTK connection was set up to a D-RTK 2 high-precision GNSS mobile station (DJI, Shenzhen, China) mounted in a tripod (Figure 2d). A Downwelling Light Sensor (DLS) 2 was positioned atop of the UAV for radiometric corrections of the multispectral sensor. Despite the primary advantage of TIR imagery being the ability to capture thermal information, TIR data from Zenmuse H20T was included in this study to explore the feasibility and effectiveness of TIR sensors in measuring geometric parameters in the context of precision viticulture.

Flight routes were planned using the DJI Pilot 2 application installed in the Matrice 300 smart controller to survey the area and surroundings of the studied vineyard (approximately 0.5 ha). Each flight was set to maintain a flight height of 50 m above the terrain, with a longitudinal overlap of 90% and a lateral overlap of 80%. The flight speeds varied for each sensor: 1.1 m s^−1^ for H20T, 1.9 m s^−1^ for L1, and 3.3 m s^−1^ for ALTUM-PT, taking approximately 12, 15, and 6 min, respectively. The planned spatial resolutions were as follows: 0.0216 m for the ALTUM-PT, 0.0444 m for the TIR imagery, 0.0172 m for the RGB imagery of the H20T sensor, 0.0136 m for the L1 LiDAR data, and 0.0158 m for the RGB imagery. Data acquisition of ALTUM-PT began at 13:20, followed by the Zenmuse L1 at 13:40 and the Zenmuse H20T at 14:00. Before acquiring the multispectral UAV data, images of a calibration reflectance panel were captured for subsequent radiometric corrections.

#### 2.2.2. TLS Data Acquisition

The TLS used in this study was the BLK360 G1 (Leica Geosystems AG, St. Gallen, Switzerland), shown in Figure 2a. This device can produce point clouds with associated RGB information for each point. It uses a high-speed time-of-flight measurement system enhanced by Waveform Digitizing (WFD) technology, offering a field of view of 360° in the horizontal plane and 300° in the vertical plane. The scanner captures up to 360,000 points per second at a wavelength of 830 and provides a 3D point accuracy ranging from 6 mm at 10 m to 8 mm at 20 m, with an acquisition range from 0.6 m up to 60 m. The RGB information is captured by a 15 MP 3-camera system, capable of generating spherical images up to 150 MP. It can be configured and operated through the Leica Cyclone FIELD 360 (Leica Geosystems AG, St. Gallen, Switzerland) software application or through a quick-release button. The BLK360 G1 is lightweight (1 kg) and can be easily deployed in field settings using a tripod.

Data acquisition for this study commenced at 11:00, using the highest resolution configuration available for BLK360 G1. Each scan took approximately nine minutes per location, including installation and stabilization time, resulting in a total scanning duration of about one hour and thirty minutes. Scanning locations were spaced seven meters apart within the same grapevine row, with targets placed 3.5 m away, covering 200 m^2^ across four rows.

### 2.3. Data Processing

#### 2.3.1. UAV Data Processing

The UAV imagery was processed using Pix4Dmapper Pro (Pix4D SA, Lausanne, Switzerland) version 4.5.6, using SfM techniques to generate dense point clouds and subsequent raster products. Data from each sensor were processed in separate projects including RGB imagery from the Zenmuse L1, RGB imagery from the Zenmuse H20T, the five multispectral bands of the ALTUM-PT, the panchromatic band of the ALTUM-PT, and the TIR imagery from H20T.

Initially, each project was subjected to processing to generate a sparse point cloud, using the full image size to extract keypoints. For image pair matching, the aerial grid or corridor configuration was selected. In all projects, except for the multispectral imagery obtained from ALTUM-PT (which used 10,000 keypoints), the target number of key points was set to automatic. The internal and external camera parameters were calibrated using the standard calibration method for projects using RGB imagery, while the alternative calibration method was used for other projects.

**Figure 3:**
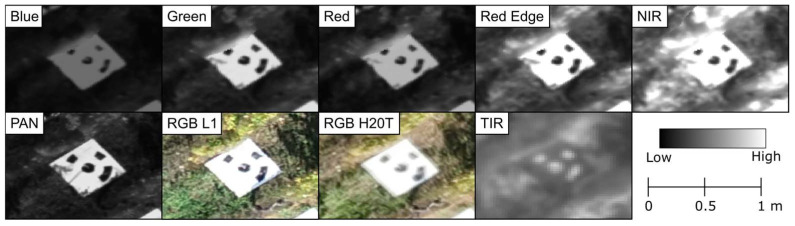
View of ground control points in the different imagery data types acquired by the sensors on the unmanned aerial vehicle. NIR: near-infrared; PAN: panchromatic; TIR: thermal infrared.

After completing this stage, GCPs were marked on the imagery (distribution shown in Figure 1a). Figure 3 provides an overview of a target used as a GCP for the various sensors. Following the identification of GCPs, the internal and external camera parameters were reoptimized to incorporate the GCP data. A dense point cloud with high point density was subsequently generated. Densification was carried out using half of the image size to compute additional 3D points (with thermal infrared using the original image size), as well as other image quarters and eight image scales. Each 3D point had to be correctly re-projected in at least three images to be considered for the dense point cloud. The resulting point clouds were exported as LAS files.

Additionally, orthorectified raster products were created, including orthophoto mosaics from the RGB imagery, Land Surface Temperature (LST) mosaics from the TIR imagery, reflectance mosaics from the multispectral and panchromatic bands, and the Normalized Difference Vegetation Index (NDVI) [71].

For the UAV LiDAR data from the Zenmuse L1, processing was conducted using DJI Terra software (version 3.9.4.). The raw data were imported and processed with a high-density setting (considering 100% of the points), assuming a flat ground surface, an iteration angle of 3°, and a distance of 0.3 m. The processed point cloud was then exported as a LAS file.

During photogrametic processing, the UAV-based RGB, multispectral, panchromatic, and TIR imagery, were aligned using GCPs. The Root Mean Square Error (RMSE) for each sensor in the surveyed vineyard plot is presented in Table 1. Overall, the planimetric errors (XY) were below 0.02 m. The multispectral imagery showed the lowest error at 0.005 m, followed by panchromatic (0.006 m) and RGB imagery from both H20T and L1 (0.008 m). The TIR imagery had the highest planimetric errors at 0.016 m. The altimetric errors (Z) ranged from 0.017 m and 0.022 m, except for TIR imagery, which had an error of 0.072 m. The spatial resolution of the resulting products varied, with panchromatic data achieving the highest resolution (0.012 m), while TIR data had the lowest (0.053 m). Data from other sensors had a spatial resolution below 0.03 m, with RGB data from L1 at approximately 0.016 m, RGB data from H20T at around 0.02 m, and multispectral data at 0.026 m.

#### 2.3.2. TLS Data Processing

TLS data were processed using Leica Cyclone REGISTER 360 PLUS (BLK Edition) version 2024.0.1, producing point clouds with RGB information. During data processing, the TLS scans were merged into a unified coordinate system for the entire point cloud. After importing the data, the “Review and Optimization operations” option was performed. This step enabled optimization, modeling, editing, error detection in scanning, and removal of unwanted points, as well as georeferencing the study area. Nine georeferenced targets (Figure 1b) were used for this purpose. The position of each scan was defined in the scanner’s coordinate systems. To align different scanning positions, it was necessary to establish the exact position and orientation of the scanner coordinate systems based on the target coordinates. Specifically, the position and orientation of the nine scanning locations were determined with reference to nine control points, thus georeferencing the TLS dataset to a fixed coordinate system. The registration accuracy of the TLS project was reported as a bundle error of 0.012 m. After processing, the point cloud was exported in LAS file format.

#### 2.3.3. Grapevine Geometrical Parameters Extraction from Point Clouds

The point clouds obtained from each sensor and imagery type of UAV-based data, including multispectral and panchromatic from Micasense ALTUM-PT, RGB and TIR from Zenmuse H20T, and LiDAR and RGB from Zenmuse L1, along with the TLS point cloud, were processed to extract several grapevine-related geometric parameters.

Each point cloud underwent a ground normalization procedure to remove the elevation variation converting altitude values into heights. The Simple Morphological Filter (SMRF) algorithm [72] was used to segment points belonging to the ground. This involved: (1) creating a surface map with the minimum altitude from the point cloud data; (2) segmenting the ground elements by separating them from other points; and (3) segmenting the original point cloud data. Altitude was transformed into height using the points identified as ground through the SMRF algorithm. An interpolation was then conducted to estimate the ground elevation for each point in the point cloud. Each point cloud was normalized by subtracting the interpolated ground altitude from the altitude of each point, creating normalized point clouds to ensure consistent comparisons across grapevine plants and different point clouds.

Once normalized, the monitored grapevines were extracted from the point clouds. This process was automated using “lasclip” function from the LAStools library (rapidlasso GmbH, Gilching, Germany) within QGIS by providing a shapefile containing polygons for each grapevine. These polygons were designed to cover a length of 1 m with a buffer of 0.8 m, encompassing the grapevine plant, its surroundings (including soil and other vegetation), and the area where the height measurements were taken in the field. This procedure allowed for the separation of points associated with each grapevine area into individual LAS files.

With each plant separated, it was possible to assess grapevine geometric parameters extracted from the point clouds generated by each sensor. In this study, the evaluated parameters included the maximum grapevine height (*H*_max_), which considers the highest point of the canopy above ground level, expressed in meters; the heights at 90th and 95th percentiles (*H*_90_ and *H*_95_, respectively) from points above the lower trellis wire, also expressed in meters; the grapevine canopy volume, in cubic meters; and grapevine projected area, in square meters.

In the individual grapevine point clouds, points below 0.30 m, which corresponds to the approximate height of the lower trellis wire in the studied vineyard, were excluded to eliminate noise points corresponding to undergrowth vegetation, which could distort the extracted metrics.

For grapevine volume estimation, a triangle mesh of the normalized point cloud was created using the alphaShape algorithm [73] for all points in X and Y dimensions with Z > 0.30 m. An alpha value (α) of 0.5 was used for UAV data, as in described in Di Gennaro and Matese [74], while α value of 0.05 was used for TLS data, following Liu et al. [75]. The same procedure was applied for grapevine projected area estimation, using the same α value. However, for area estimation, only the X and Y dimensions from points with Z > 0.30 m were considered to create a polygon encompassing the entire grapevine canopy.

### 2.4. Data Analysis

The point clouds produced from each sensor’s data went through a qualitative assessment. This process involved visually inspecting the point clouds to identify similarities and differences across datasets and to derive some metrics related to their point density. Additionally, the distribution of the estimated grapevine geometric parameters within the point cloud data was examined, including considerations of the orthorectified products generated by each sensor.

A statistical analysis of the data extracted for each grapevine was conducted using R software [76] version 2023.12.0 and IBM SPSS Statistics version 28.0.1.0 (IBM Corp., Armonk, NY, USA). Key discriminative statistics, such as mean, minimum, maximum, Standard Deviation (SD), and Coefficient of Variation (CV), were calculated for each measurement tool. To assess the reliability and validity of the extracted geometrical variables, linear correlations among all measured parameters were analyzed. The evaluation of the results was supported by statistical parameters, including RMSE, coefficient of determination (*R*^2^), and statistical significance, using the R package “lmodel2” [77] version 1.7-3 determined through the F-test. The findings were visually represented using box plots and correlation matrices using “ggplot2” [78] R package version 3.4.4. Paired correlations of means were examined using Pearson’s test (*r*), following Mukaka’s analysis [79]. All reported intervals reflect a 95% confidence interval.

The normality of the data was assessed using the Kolmogorov–Smirnov and Shapiro–Wilk tests, while the assumption of homogeneity of variance was evaluated using Levene’s test. In cases where the assumptions of normality and homogeneity of variance were not satisfied (*p* < 0.05), a one-way analysis of variance (ANOVA) was conducted to determine if there were significant discrepancies in the geometric parameters of the grapevines between the different measurement methods. To improve the reliability of the findings and account for deviations from normality in the sample distribution, bootstrapping procedures (1000 resamples; 95% CI BCa) were employed, resulting in a 95% confidence interval for the differences between the means [80]. Given the presence of heterogeneity of variance, Welch’s correction was applied, and post-hoc analysis was performed using the Games-Howell technique [81].

## 3. Results

### 3.1. Data Characterization

**Figure 4:**
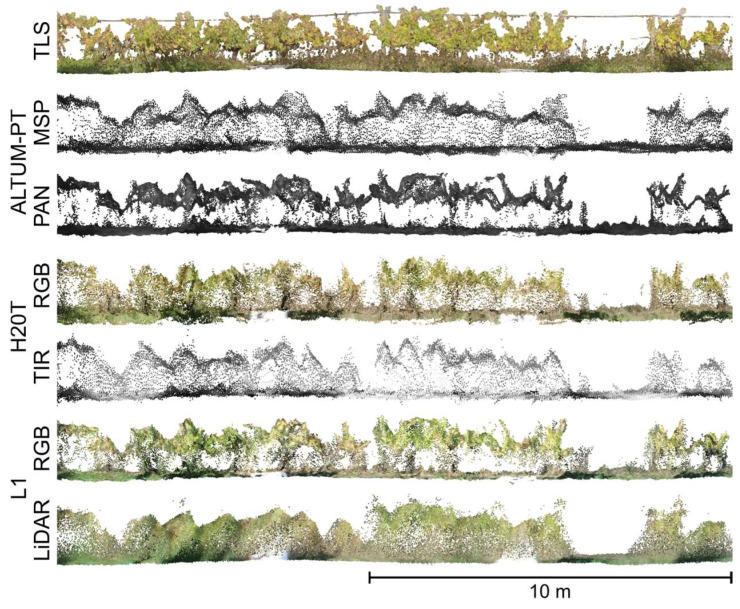
Comparative view of the point clouds generated from the different sensors in part of a grapevine row in the study area. TLS: terrestrial laser scanner; MSP: multispectral; PAN: panchromatic; TIR: thermal infrared.

The point cloud data from the different sensors presented distinct point density distributions. For the entire vineyard plot, the highest point density among the UAV-based point clouds was observed in the LiDAR data, with 3123 points per m^3^. Of the more than 17.5 million points registered for vineyard plots, 98.97% were first return points, 1.02% were second return points, and 0.01% were third return points. This was followed by the RGB imagery from H20T (2678 points per m^3^), the panchromatic imagery of ALTUM-PT (2241 points per m^3^), and the RGB imagery from L1 (2089 points per m^3^). The multispectral and TIR imagery had lower point densities at 535 and 466 points per m^3^, respectively. In contrast, the TLS point cloud achieved an average of 199,718 points per m^3^. Figure 4 presents a lateral perspective of part of one row of each generated point cloud, illustrating the distribution of points along the Z-axis.

**Figure 5:**
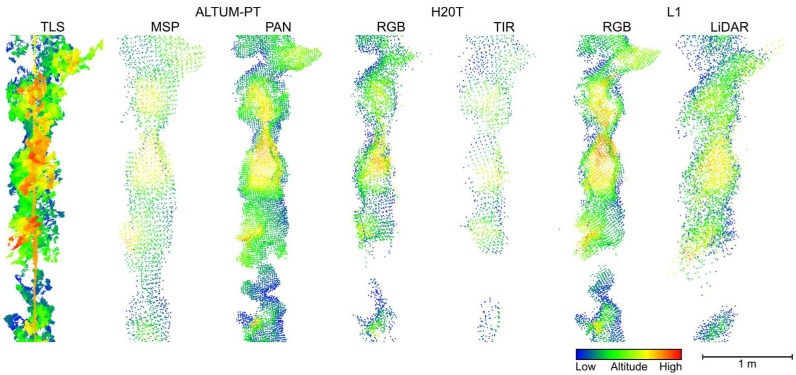
Top perspective of the point distribution along the grapevine canopy from point clouds generated by different sensors in part of a grapevine row. TLS: terrestrial laser scanner; MSP: multispectral; PAN: panchromatic; TIR: thermal infrared.

Regarding the average number of points within the grapevine canopies, the photogrammetric processing of the panchromatic data produced an average of 2451 points, L1 RGB data had 2220 points, and the L1 LiDAR data showed 1557 points. The RGB imagery from H20T produced an average of 1253 points, the multispectral imagery had 626 points, and the TIR imagery had an average of 328 points within the grapevine canopy. The point density from the TLS sensor was approximately one thousand times higher than that of the UAV sensors, with an average of 751,780 points. These differences are illustrated in Figure 5, which displays the point distribution and density of the grapevine canopy along part of a row.

**Figure 6:**
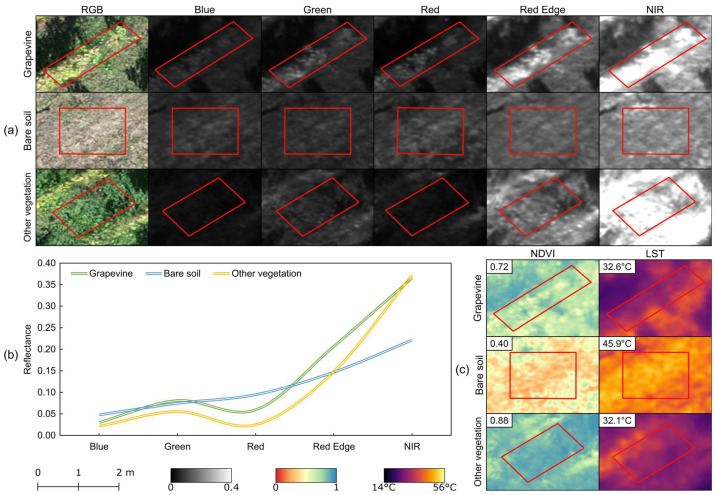
Spectral and thermal behavior of acquired data in different vineyard elements (grapevine, bare soil, other vegetation): (**a**) visual representation of the raster products, (**b**) reflectance in the five spectral bands, and (**c**) mean values of normalized difference vegetation index (NDVI) and land surface temperature (LST).

In addition to generating point clouds, UAV sensor imagery can also create raster products. Examples of these products are presented in Figure 6, including orthophoto mosaics derived from RGB imagery and reflectance from multispectral bands (Figure 6a). The orthophoto mosaic is useful for visual inspection of the vineyard, while the multispectral data provides valuable information for detailed analysis. The spectral bands offer insights into different elements within the vineyard, particularly parameters related to grapevines. The multispectral sensor used in this study captures five distinct bands, enabling the characterization of elements such as bare soil, grapevines, and other vegetation (Figure 6b). These spectral bands are also used to compute vegetation indices, such as NDVI, and provide temperature-related information from TIR imagery (Figure 6c).

**Figure 7:**
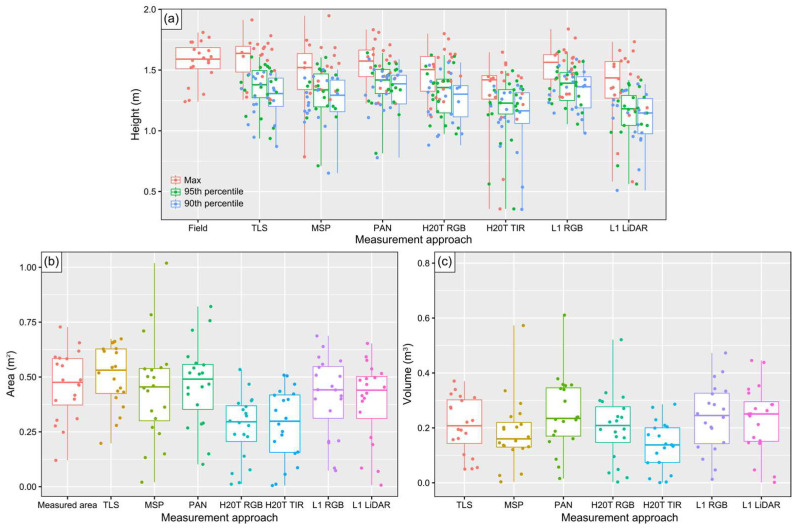
Box plot distribution of geometrical parameters of the analyzed grapevines obtained using different measurement methods: (**a**) height metrics, (**b**) projected area, and (**c**) canopy volume.

A statistical analysis related to the dispersion of the height variables of the evaluated grapevines using different measurement tools is summarized in Table 2. Figure 7 presents the distribution of different metrics of the analyzed grapevines across all sensors and ground truth measurements.

For *H*_max_ (Figure 7a), considerable variation among data from the evaluated sensors is evident. The SD was relatively consistent across sensors, ranging from 0.16 m for the L1 RGB data to 0.32 m in the H20T TIR data. CVs showed greater variability, ranging from 10.3% to 24.3% among these sensors. The TLS data closely matched the field data, with a mean of 1.59 m (+0.02 m compared to field data), low SD (0.18 m, +0.01 m compared to field data), and consistent distribution (CV = 11.7%). The panchromatic, L1 RGB, multispectral, and H20T RGB point cloud data also had mean values close to field values, with differences of less than 0.05 m: 0.02 m for panchromatic and 0.04 m for L1 RGB, with 0.09 m for multispectral and RGB H20T. Low SD and CV values were observed, except for multispectral data (SD = 0.24 m and CV = 16.25). The H20T TIR sensor had the lowest average *H*_max_ value (1.30 m) and the highest SD (0.32 m), with a range from 0.36 to 1.65 m (CV = 24.32%), showing the greatest data dispersion around the mean.

For *H*_90_ and *H*_95_ (Figure 7a), the data derived from panchromatic imagery presented the highest mean values for both variables (1.38 m for *H*_95_ and 1.33 m for *H*_90_), with an SD of 0.19 m and CV of 13.89% and 13.61%, respectively. This was followed by the L1 RGB data (*H*_95_ = 1.37 m, *H*_90_ = 1.32). Similar values are observed for the TLS data (1.34 and 1.27, respectively for *H*_95_ and *H*_90_). These sensors also showed lower SD values, indicating less dispersion compared to other sensors. In contrast, the TIR imagery and L1 LiDAR data showed the lowest mean values for both percentiles (*H*_90_ = 1.10 m for both sensors, *H*_95_ = 1.15 m for LiDAR data, and *H*_95_ = 1.16 m for TIR data). The TIR data had greater dispersion, with *H*_90_ values ranging from 0.35 m and 1.38 m, an SD of 0.27 m, and a CV of 24.89%. For *H*_95_, the range was between 0.36 m and 1.49 m, with an SD of 0.28 m and a CV of 24.42%.

Regarding the grapevine projected area (Figure 7b and Table 3), the TLS point cloud data closely matches the measured area, with the highest mean across sensors (0.51 m^2^, +0.05 m^2^ compared to the measured area), and a lower standard deviation (0.14 m^2^, +0.01 m^2^ compared to the measured area), ranging from 0.20 m^2^ to 0.67 m^2^, with a more consistent data distribution compared to other sensors (CV = 28.07%). The panchromatic, multispectral, and L1 RGB point clouds also show mean values close to measured values, with differences of less than 0.05 m^2^. The H20T RGB and H20T TIR data had the lowest mean projected areas (0.27 and 0.28 m^2^, respectively) and the highest variation (CV = 54.04% and 57.83%), indicating the greatest data dispersion around the mean.

For the grapevine volume (Figure 7c and Table 3), variation among sensors was observed. The mean grapevine volume ranged between 0.136 m^3^ in the TIR data to 0.251 m^3^ in the panchromatic data. The SD across these sensors ranged from 0.085 m^3^ to 0.135 m^3^, with CVs ranging from 41.66% in the TLS data to 67.45% in the multispectral data. Among the UAV-based sensors, the panchromatic point cloud registered the highest mean values (0.251 m^3^; SD = 0.135 m^3^; CV = 53.80%), followed by the L1 RGB and LiDAR data, which showed mean values of 0.238 m^3^ and 0.232 m^3^, respectively, with corresponding SDs of 0.123 m^3^ and 0.124 m^3^, and CVs of 51.46% and 53.28%. These values were close to those from the TLS point cloud results (0.232 m^3^; SD = 0.097 m^3^; CV = 41.66%). The RGB data from the H20T showed a mean volume of 0.203 m^3^, while the multispectral point cloud had 0.184 m^3^. The H20T TIR data had the lowest mean volume at 0.136 m^3^, and a CV of 62.73%.

Normality distribution tests (Kolmogorov–Smirnov and Shapiro–Wilk) indicated that the variables did not follow a normal distribution (*p* < 0.05). Additionally, Levene’s test revealed a lack of homogeneity of variance between the variables (*p* < 0.05). Due to the violation of these assumptions, a one-way ANOVA could not be applied. Instead, the Games-Howell non-parametric post-hoc test was conducted and interpreted using bootstrapping procedures. This analysis revealed significant differences between the sensors for the variables examined (*p* < 0.05). The results are presented in Table 2 and Table 3. For *H*_max_, the TLS, multispectral, panchromatic, H20T RGB, and L1 RGB data showed statistically similar averages. However, the L1 LiDAR data demonstrated significant similarity to the multispectral, H20T RGB, and TIR point cloud data. For *H*_90_, the TLS, multispectral, panchromatic, H20T RGB, and L1 RGB point cloud data had statistically similar averages, whereas the H20T TIR data showed significant similarity with the multispectral, H20T RGB and L1 LiDAR. For *H*_95_, the TLS, multispectral, panchromatic, H20T RGB, and L1 RGB data showed statistically similar averages, while the H20T TIR and L1 LiDAR data only showed significant similarity to each other. Regarding grapevine projected area, the measured area and the TLS, multispectral, panchromatic, and L1 RGB data exhibited statistically similar averages, while the L1 LiDAR sensor demonstrated significant similarity to the measured area, multispectral, panchromatic, and L1 RGB data. The H20T RGB and H20T TIR data demonstrated only statistically similar averages to each other. Finally, for grapevine canopy volume, the TLS, multispectral, panchromatic, H20T RGB, L1 RGB, and L1 LiDAR data demonstrated statistically similar averages, except for H20T TIR data, which exhibited significant similarity to multispectral and H20T RGB.

### 3.2. Grapevine Geometric Parameters

Correlations between the geometric parameters extracted from the point cloud data of each sensor were established to verify the accuracy of the point clouds in representing the structure of the grapevine canopy. Table 4 compares the results obtained by the different sensors, including performance metrics such as the correlation coefficient (*r*), coefficient of determination (*R*^2^), and RMSE for the geometric parameters of grapevines.

**Figure 8:**
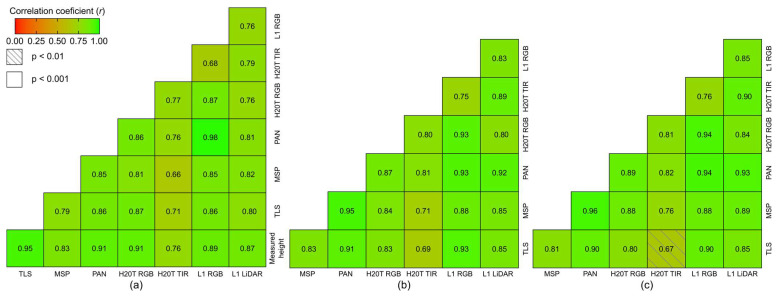
Correlation matrix between height variables obtained from different sensors for (**a**) maximum height and field-measured height; (**b**) height at the 95th percentile; and (**c**) height at the 90th percentile. The diagonal line is intentionally omitted.

The correlation matrix reported in Figure 8a shows that the correlation between the field-measured height values and *H*_max_ is strong to very strong and significant (*r* > 0.7; *p* < 0.001) for all sensors. Using the measured parameters from the TLS point cloud as a reference to suppress field values for *H*_95_ (Figure 8b) and *H*_90_ (Figure 8c), the results indicate that all point clouds were able to correctly represent these variables, showing moderate to very strong correlation across all UAV sensors (*r* > 0.5; *p* < 0.01).

For grapevine *H*_max_, the TLS data exhibited a strong, significant correlation (*r* = 0.95) and the highest *R*^2^ (0.90), indicating a strong linear relationship between the data captured by the sensor and actual grapevine height. The RMSE was also low (0.027 m). Among the UAV-based sensors, the panchromatic data showed significant results relative to ground-truth data (*r* = 0.91), the highest *R*^2^ (0.83), and an RMSE of 0.025 m. This was followed by the H20T RGB data (*r* = 0.91, *R*^2^ = 0.83), with an RMSE of 0.081 m; the L1 RGB (*r* = 0.89, *R*^2^ = 0.79) and LIDAR (*r* = 0.87, *R*^2^ = 0.76) data with RMSE values of 0.038 m and 0.129 m, respectively, and the multispectral data (*r* = 0.83, *R*^2^ = 0.70), with an RMSE of 0.084 m. The lowest correlation values were achieved by the TIR data (*r* = 0.76, *R*^2^ = 0.58) with the highest RMSE (0.147 m). Among UAV sensors, the highest correlations were between the panchromatic and L1 RGB data (*r* = 0.98), while the lowest correlation was between TIR imagery and multispectral data (*r* = 0.66).

When correlating the UAV-based data with the parameters extracted from the TLS point cloud for *H*_95_ (Figure 8b) and *H*_90_ (Figure 8c), the panchromatic point cloud data showed very strong, significant correlations for both percentiles, with *r* = 0.91 and 0.90 (*p* < 0.001), for *H*_95_ and *H*_90_, respectively. Similarly, the L1 RGB data demonstrated strong correlations for both percentiles, with *r* = 0.93 and 0.89 (*p* < 0.001), respectively. The LiDAR data from the L1 sensor also showed a good correlation, with *r* = 0.85 (*p* < 0.001) for both percentiles. The lowest correlations were found for the H20T TIR data in both percentiles. Among the UAV sensors, the highest correlations were between panchromatic and multispectral data (*r* = 0.95 for *H*_95_ and *r* = 0.96 for *H*_90_; *p* < 0.001). The lowest correlation for *H*_90_ was between TIR with L1 RGB and multispectral data (*r* = 0.76, *p* < 0.01). For *H*_95_, the lowest correlation was between TIR and multispectral data (*r* = 0.71, *p* < 0.01).

**Figure 9:**
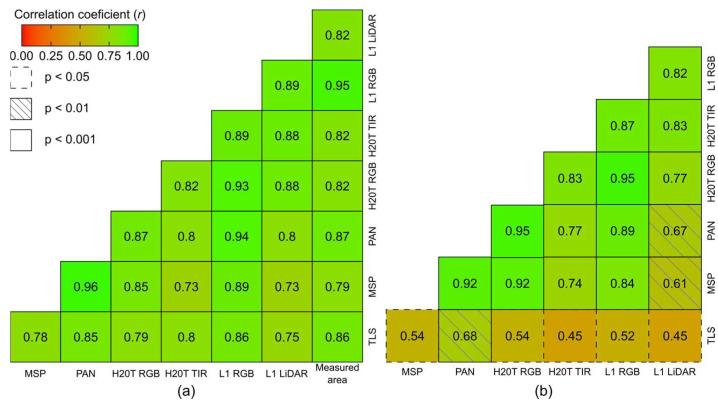
Correlation matrix for grapevine projected area (**a**) and canopy volume (**b**) from the different sensors. The diagonal line is intentionally omitted.

In the analysis of the projected grapevine area (Table 4), the TLS data showed a strong, significant correlation (*r* = 0.86, *p* < 0.001; *R*^2^ = 0.74). Among UAV-based sensors, the L1 RGB data demonstrated a very strong and significant correlation (*r* = 0.95, *p* < 0.001) and the best *R*^2^ (0.89) with the measured area, despite having a slightly higher RMSE (0.048 m^2^) compared to the TLS and panchromatic data (RMSE = 0.042 m^2^). The panchromatic data followed closely (*r* = 0.87, *p* < 0.001), along with the H20T RGB, H20T TIR, and L1 LiDAR (*r* = 0.82, *p* < 0.001). The lowest correlation was observed in the multispectral data (*r* = 0.79, *p* < 0.001). Among UAV sensors (Figure 9a), the highest correlation was between multispectral and panchromatic data (*r* = 0.96, *p* < 0.001), while the lowest correlation was between multispectral, LiDAR and TIR data (*r* = 0.73, *p* < 0.001). For grapevine canopy volume (Figure 9b), there were disparities among the sensors. The panchromatic point cloud data showed the highest correlation coefficient with TLS data (*r* = 0.68, *p* < 0.01), while the TIR data exhibited the lowest overall correlation (*r* = 0.68, *p* < 0.01). The multispectral data displayed a lower correlation (*r* = 0.45, *p* < 0.05). Among UAV sensors, the highest correlations were found for the H20T RGB data with panchromatic and L1 RGB data (*r* = 0.95, *p* < 0.001). The lowest observed correlation was between L1 LiDAR and multispectral data (*r* = 0.61, *p* < 0.01).

## 4. Discussion

This study aimed to evaluate and compare the effectiveness of various sensors and data acquisition methods in characterizing the geometric parameters of grapevines. The results indicate significant differences in point density and accuracy among the sensors, which directly impacts the precision of the geometric measurements. Detecting the variability within vineyards is crucial for PV, as it enables the automation of operations, optimization of chemical inputs, and mitigation of environmental impacts [34]. This assessment is essential for differentiating operations such as pruning, harvesting, fertilization, and crop protection [18]. As the geometric characteristics are directly related to plant vigor [41,82,83], this study compared point clouds from multispectral, panchromatic, RGB, TIR, and LiDAR UAV data, as well as TLS point clouds, were compared to evaluate the height variables (*H*_max_, *H*_90_, *H*_95_), grapevine projected area, and grapevine volume. Measurements of maximum height (*H*_max_) and projected area were taken as a reference to assess the ability of the point clouds derived from each sensor and data type for the characterization of grapevine geometric parameters [16,34,36].

As expected from previous studies [34,67], the TLS point cloud had a higher point density compared to UAV data. This higher density can be attributed to the static nature of the TLS sensor, which enables 360-degree scans at each collection point, resulting in superior resolution and detail. This is reflected in the average number of grapevine points (Figure 5). The UAV sensors showed varying point densities with LiDAR data generally providing a higher density than imagery subjected to photogrammetric processing, which depends on the image resolution [84,85].

The *H*_max_ values obtained from point clouds, when compared to the field measurements (Table 2 and Table 4), were found to be reliable for automatically calculating grapevine *H*_max_ (*r* > 0.7; *p* < 0.001). The TLS point cloud data provided the highest average and the lowest standard deviation (Table 2) and the highest correlations (Table 4). Although the multispectral and TIR point clouds showed lower correlations with field measurement, multispectral data can still be indicative of vigor and grapevine height [21,86]. The RGB, panchromatic, and LiDAR point clouds also demonstrated good correlations with *H*_max_, with the panchromatic data showing the lowest RMSE, improving on previously reported results for height estimation from UAV data [52]. The visual analysis of Figure 4 supports these findings, where the TLS point cloud shows a more detailed vertical representation compared to the UAV-based point clouds, which appears smoother, despite the relatively accurate representation at the top part provided by panchromatic and RGB point clouds [87,88,89]. Similar trends have been reported in other studies comparing UAV and TLS data in vineyards. Pagliai et al. [34] reported a mean of 1.04 m and a CV of 13% obtained using a mobile TLS and a mean of 1.07 m and a CV of 12% with data from UAV RGB imagery. In Torres-Sánchez et al. [31], the mean maximum height of grapevines obtained was 2.22 ± 0.25 m and 2.04 ± 0.32 m for mobile TLS and UAV RGB data, respectively. In Escolà et al. [67], the mean heights of 2.19 m and 1.92 m (*H*_max_ and *H*_90_) for mobile TLS and 2.09 m and 1.99 m (*H*_max_ and *H*_90_) for UAV RGB data. The findings of this study showed superior performance when compared to previous studies that compared real measurements with mobile TLS estimates [50,90] (Table 5). These discrepancies may be attributed to the continuous structure of grapevines, which complicates manual ground truth measurements. However, the results are consistent with those reported by Rinaldi et al. [48].

Given the high accuracy of the TLS estimates and their greater similarity to actual values in the *H*_max_ estimates, the other geometric height parameters (*H*_95_ and *H*_90_) were compared with the TLS-based dataset (Figure 8b,c), which can serve as ground truth reference, as done in other studies [91]. This provides greater credibility to the use of TLS as a reference for geometric parameters. The point clouds obtained from the UAV data demonstrated strong and significant correlations with *H*_95_ and *H*_90_ extracted from the TLS point clouds (*r* > 0.8; *p* < 0.001), except for the H20T TIR, which had a slightly lower correlation. The panchromatic and L1 RGB point clouds exhibited the strongest correlations, indicating that despite variations in performance, all UAV sensors generally provide reliable height data that is consistently correlated with TLS measurements, similar to findings from similar studies [34,67].

Regarding the grapevine projected area (Figure 9a), the TLS data exhibited the highest mean value (Table 3), with a non-significant difference of 10.9% compared to the measured area. The area estimated from the panchromatic point cloud closely aligns with field data. However, all sensors, except H20T RGB and H20T TIR, were statistically equivalent to the measured average value (*p* < 0.05). The results, consistent with the correlations, indicate that both TLS and UAV sensors can estimate the projected grapevine area accurately, with the L1 RGB sensor showing the highest correlation (*r* = 0.95, *p* < 0.001) with the measured average. Other studies have revealed an *R*^2^ = 0.78 (*p* < 0.001) for this parameter [31].

The grapevine volume data (Figure 9b and Table 3) estimated from the sensors showed some disparities. The TLS point cloud had the lowest variance, followed by the L1 LiDAR. In contrast, the TIR data provided less satisfactory results in terms of precision and consistency. These findings are consistent with those of Torres-Sánchez et al. [31], who reported values of 0.24 ± 0.06 m^3^ and 0.19 ± 0.06 m^3^ from a mobile TLS and UAV, respectively. However, the volume calculated by Torres-Sánchez et al. [31] was based on the canopy height model (CHM), assuming that all space between the ground and the canopy top was occupied by the canopy. Meanwhile, Pagliai et al. [34] found a mean volume of 0.40 m^3^ and a variation of 15% using a mobile TLS, and 0.59 m^3^ with UAV, showing 22% variation. The estimated volumes in this study were lower than those reported by Escolà et al. [67], who calculated volume as the sum of the cross-sectional areas multiplied by the section length. This highlights the need to explore and compare different approaches for grapevine volume estimation [75], complementing these analyses with field-measured volume validation [52]. When comparing grapevine volume estimates from TLS and UAV sensors, strong and significant correlations were observed (*r* > 0.7, *p* < 0.05) for most sensors, except for multispectral data, which still showed a significant correlation (*p* < 0.01). The panchromatic data exhibited the highest correlation (Figure 9b). Among the UAV sensors, more favorable correlations were observed, although the L1 LiDAR data demonstrated a moderate correlation (*r* < 0.7) with multispectral and panchromatic data. Interestingly, a close similarity was found between UAV TIR and LiDAR data for volume estimation (*r* = 0.83, *p* < 0.001), similar to Buunk et al. [91]. The observed correlations in other studies on grapevine volume estimation are summarized in Table 5. The findings contrast with those of Chakraborty et al. [90], who reported a strong correlation between volume estimates from mobile TLS and canopy surface area from UAV multispectral imagery. Petrović et al. [44] demonstrated a strong correlation between UAV RGB data and mobile TLS volume measurements. Tumbo et al. [92] found that laser sensors provided more accurate canopy volume estimates than ultrasonic sensors due to their higher resolution (*R*^2^ > 0.85, RMSE < 2.15 m^3^).

The underestimation of heights by TIR and L1 LiDAR sensors, variations in the points distribution (notorious in Figure 5), and greater variability in UAV sensor results for projected area and volume estimation (Figure 9) can be attributed to the growth behavior of grapevines. Grapevines develop thin, long branches intertwined with individual leaves in the upper canopy. These thin parts are challenging to detect in aerial images and may not be fully reconstructed using UAV data. This is clear in the generated point clouds, where grass is less apparent in UAV data compared to the TLS point cloud (Figure 4), and details in thin branches are lacking (Figure 5). Additionally, long branches often hang beside the vineyard rows, significantly contributing to the projected area and volume [31,68,93]. This phenomenon was also observed by Torres-Sánchez et al. [31], who reported greater discrepancies in area estimates in vineyards compared to pear and peach orchards, which lack lateral branches. To address canopy height underestimation in UAV methodology, increasing image spatial resolution may be beneficial [31]. Andújar et al. [40] observed that mobile TLS data resulted in higher canopy volume values than UAV data in vineyard analysis. However, Pagliai et al. [34] found the highest volume estimates from the UAV workflow in a comparison with mobile TLS and ground-based photogrammetry. This discrepancy was attributed to noise in the point cloud and difficulty in accurately isolating grapevines. Moreover, area and volume estimates derived from the TLS can be less precise than height estimates [44,48]. This may be due to overlapping errors from scanning both sides of the plants, leading to error accumulation. The high point density and presence of grass can also overestimate the area and volume values due to potential noise. However, in this study, steps were taken to mitigate these errors during data acquisition and processing before the extraction of the geometrical parameters.

In this study, the UAV imagery was captured at a nadir angle, unlike other studies that used oblique angles [67], leading to lower estimates. This conclusion is supported by the highest correlation found between *H*_max_ in the point cloud derived from TLS and field measurements, as the TLS scanning was oriented laterally between rows. This is in line with previous research indicating that the combination of nadir and oblique angles results in more accurate 3D analysis [91,94,95]. Another factor complicating the use of UAV-based imagery to determine the ground surface below the canopy is the presence of tall grass (Figure 4), which results in a noisier ground surface.

The results reported in this study are valuable for PV applications, providing insights into the optimal use of stationary TLS or UAV sensors for geometric parameter estimation, which is crucial for vineyard monitoring and decision support systems. Future research should prioritize data collection across different phenological stages and vineyard settings [60], including steep-slope vineyards and various grape varieties with different training systems. It should also incorporate additional parameters such as canopy length and width, trunk diameter, as well as NDVI and LAI as biophysical parameters in the analyses. Moreover, the effects of UAV-based imagery parameters, such as spatial resolution, flight height, imagery overlap, and camera angle, on grapevine parameter estimates, should be assessed across different periods and grapevine varieties. This will facilitate the optimization of UAV imagery acquisition for geometric parameter extraction [66,96,97,98,99].

Highlighting the advantages and limitations of TLS and UAV systems is crucial for understanding their applications in different scenarios. UAV-based systems offer large-scale measurement capabilities due to their ability to conduct measurements at relatively lower flight heights, resulting in a wide field of view [34]. In this study, UAV data acquisition enabled the rapid surveying of the entire vineyard plot, whereas TLS data acquisition took 1 h and 30 min for a smaller portion of the plot (Figure 1b), potentially limiting the use of TLS for larger areas [41]. Therefore, UAV data acquisition allows for rapid mapping of large areas, while stationary TLS is limited to smaller areas. TLS requires sufficient coverage and overlap to ensure accuracy, minimize data gaps, and improve the overall quality of the 3D model generated from the data [100]. In contrast, mobile TLS systems enables can be adapted to agricultural vehicles, enabling automatic data collection during field operations [31,41,42,83].

However, studies comparing the performance of static TLS and mobile TLS for detecting trees and estimating dendrometric parameters in forest areas [101,102] have demonstrated that, despite the lower accuracy of mobile TLS, the results from both systems are comparable and do not show significant differences in estimates. This suggests that the parameter extraction methodology used in this study is applicable to vineyard data acquired using mobile TLS systems, thereby optimizing the efficiency of this sensor technology. Nevertheless, the use of TLS, primarily mobile TLS, can be challenging in sloped vineyards and uneven terrains [103]. On the other hand, while UAVs do not face these challenges, they require trained personnel and must comply with specific legal regulations [34]. A limitation of UAV data acquisition is the potential information loss, such as difficulties in reconstructing thin lateral branches [31]. Among the evaluated UAV sensors, the ALTUM-PT showed high accuracy in estimating geometrical parameters, particularly when using the panchromatic band, which was effective for measuring height and grapevine projected area. This is significant, as the sensor captures multispectral data (Figure 6), crucial for diverse vineyard-related tasks, along with thermal infrared imagery. In contrast, the point cloud generated from TIR imagery demonstrated a lower performance in evaluating the assessed parameters due to its lower spatial resolution. To mitigate such errors, data fusion techniques can be employed, either during the photogrammetric processing by integrating different imagery types (e.g., RGB and TIR) or by projecting data from lower-resolution sensors into high-resolution point clouds [104,105]. Moreover, UAV-based RGB point cloud data can be integrated with lower-resolution raster data, such as multispectral and TIR imagery, to isolate grapevine information and assist in mapping vineyard variability [106]. Additionally, data fusion techniques involving ground and aerial data, along with SfM outcomes and TLS point clouds, can be explored to achieve precise and accurate results. This particularly valuable in complex topographic environments, such as agricultural terraces [107].

## 5. Conclusions

This study compared point cloud data calculated from TLS and UAV LiDAR and generated from different UAV sensors subjected to photogrammetric processing to evaluate geometric parameters such as height, projected area, and volume in grapevines. The goal was to establish a benchmark for precise vineyard management by assessing the available tools for grapevine geometry analysis. Detecting variability within vineyards is essential for PV, enabling the automation of operations and optimization of inputs while reducing environmental impacts. The results confirm the feasibility of using TLS and UAV-derived point clouds for automatically calculating grapevine geometric parameters. TLS point clouds provided the most accurate measurements, closely matching field data and serving as a reliable reference. Among UAV sensors, the panchromatic imagery from ALTUM-PT was the most suitable data for height estimates, followed by RGB imagery from Zenmuse L1 and H20T. For the projected area, RGB images from Zenmuse L1 were the most suitable, followed by panchromatic images from ALTUM-PT and Zenmuse L1 LiDAR data. In volume estimation, the panchromatic imagery from ALTUM-PT showed a good performance, followed by RGB images from Zenmuse L1 and L1 LiDAR data. While UAV sensors show effectiveness, especially when correlated with TLS data, variations among sensors underscore the importance of careful selection based on specific application needs and environmental conditions.

TLS-based point cloud data offers greater precision and reliability due to its proximity to the grapevines and detailed characterization of both lateral views of the plants. However, it is less efficient in terms of data acquisition and processing time. The choice of the best system ultimately depends on a range of factors such as environmental conditions, equipment availability, and survey objectives. TLS is preferable for detailed information, while UAVs are more suitable for covering extensive areas.

This study also highlights the importance of understanding the limitations and factors influencing sensor data accuracy in vineyard assessments. Differences in volume estimates among sensors highlight challenges in UAV-based measurements. Factors such as flight angle and interference from ground vegetation can affect the accuracy of geometric parameter estimates. Future research should focus on data collection across various phenological stages, different vineyard settings (e.g., steep slopes), and grape varieties with varying training systems. Incorporating additional biophysical parameters, and minimizing biases will enhance TLS and UAV data acquisition and processing techniques in PV. Furthermore, future studies should assess the impact of optimizing UAV flight parameters on the estimation of geometric parameters.

## Figures and Tables

**Table 1:** Planimetric (XY), altimetric (Z), and overall (XYZ) Root Mean Square Error (RMSE) of UAV imagery alignment of the imagery from each sensor during photogrammetric processing.

Sensor	Imagery Type	RMSE XY (m)	RMSE Z (m)	RMSE XYZ (m)	Spatial Res. (m)
ALTUM-PT	Multispectral	0.005	0.021	0.013	0.0262
	Panchromatic	0.006	0.017	0.011	0.0124
Zenmuse L1	RGB	0.008	0.018	0.012	0.0158
Zenmuse H20T	RGB	0.008	0.022	0.014	0.0197
	Thermal infrared	0.016	0.072	0.044	0.0527

**Table 2:** Statistics of the height variables of the grapevines for the different measurement tools. Mean values with the same letters do not present significant differences (*p* < 0.05). SD: standard deviation; CV: coefficient of variation; MSP: multispectral; PAN: panchromatic; TIR: thermal infrared.

Sensor	Data Type	Min.	Max.	Mean	SD	CV (%)
**Maximum height (m)**						
Measured		1.24	1.81	1.57 ^a^	0.17	10.84
BLK360	TLS	1.26	1.91	1.59 ^a^	0.18	11.67
ALTUM-PT	MSP	0.79	1.95	1.48 ^a,b^	0.24	16.25
	PAN	1.22	1.83	1.55 ^a^	0.18	11.35
Zenmuse H20T	RGB	1.12	1.80	1.48 ^a,b^	0.19	13.11
	TIR	0.36	1.65	1.30 ^c^	0.32	24.32
Zenmuse L1	RGB	1.28	1.84	1.53 ^a^	0.16	10.34
	LiDAR	0.58	1.73	1.37 ^b,c^	0.29	21.05
**90th percentile of height (m)**						
BLK360	TLS	0.87	1.48	1.27 ^a^	0.18	14.32
ALTUM-PT	MSP	0.65	1.50	1.25 ^a,b^	0.20	15.74
	PAN	0.78	1.59	1.33 ^a^	0.19	13.89
Zenmuse H20T	RGB	0.88	1.56	1.25 ^a,b^	0.21	16.64
	TIR	0.35	1.38	1.10 ^b,c^	0.27	24.89
Zenmuse L1	RGB	0.98	1.59	1.32 ^a^	0.16	12.31
	LiDAR	0.51	1.39	1.10 ^c^	0.23	20.63
**95th percentile of height (m)**						
BLK360	TLS	0.94	1.61	1.34 ^a^	0.19	13.8
ALTUM-PT	MSP	0.71	1.60	1.31 ^a^	0.20	15.52
	PAN	0.81	1.64	1.38 ^a^	0.19	13.61
Zenmuse H20T	RGB	0.97	1.62	1.31 ^a^	0.20	15.41
	TIR	0.36	1.49	1.16 ^b^	0.28	24.42
Zenmuse L1	RGB	1.05	1.65	1.37 ^a^	0.16	11.4
	LiDAR	0.56	1.42	1.15 ^b^	0.22	19.26

**Table 3:** Statistics of the projected area and canopy volume of the grapevines for the different measurement tools. Mean values with the same letters do not present significant differences (*p* < 0.05). SD: standard deviation; CV: coefficient of variation; MSP: multispectral; PAN: panchromatic; TIR: thermal infrared.

Sensor	Data Type	Min.	Max.	Mean	SD	CV (%)
**Projected area (m^2^)**						
Measured		0.12	0.73	0.46 ^a,b^	0.15	33.46
BLK360	TLS	0.20	0.67	0.51 ^a^	0.14	28.07
ALTUM-PT	MSP	0.02	1.02	0.44 ^a,b^	0.23	52.57
	PAN	0.10	0.82	0.47 ^a,b^	0.19	39.99
Zenmuse H20T	RGB	0.01	0.53	0.27 ^c^	0.15	54.04
	TIR	0.01	0.51	0.28 ^c^	0.16	57.83
Zenmuse L1	RGB	0.07	0.69	0.41 ^a,b^	0.18	42.93
	LiDAR	0.01	0.65	0.39 ^b^	0.18	46.82
**Canopy volume (m^3^)**						
BLK360	TLS	0.055	0.372	0.232 ^a^	0.097	41.66
ALTUM-PT	MSP	0.004	0.573	0.184 ^a,b^	0.124	67.45
	PAN	0.016	0.611	0.251 ^a^	0.135	53.8
Zenmuse H20T	RGB	0.003	0.521	0.203 ^a,b^	0.125	61.34
	TIR	0.001	0.286	0.136 ^b^	0.085	62.73
Zenmuse L1	RGB	0.013	0.473	0.238 ^a^	0.123	51.46
	LiDAR	0.002	0.445	0.232 ^a^	0.124	53.28

**Table 4:** Statistical parameters (correlation coefficient (*r*), coefficient of determination (*R*^2^), root mean square error (RMSE)) of the maximum height and area of the measured grapevines with metrics derived from each sensor. TLS: terrestrial laser scanner; TIR: thermal infrared.

Sensor	Data Type	Maximum Height (m)			Area (m^2^)		
		*r*	*R* ^2^	RMSE	*r*	*R* ^2^	RMSE
BLK360	TLS	0.95	0.90	0.027	0.86	0.74	0.042
ALTUM-PT	Multispectral	0.83	0.70	0.084	0.79	0.63	0.072
	Panchromatic	0.91	0.83	0.025	0.87	0.76	0.042
Zenmuse H20T	RGB	0.91	0.83	0.081	0.82	0.66	0.165
	TIR	0.76	0.58	0.147	0.82	0.67	0.143
Zenmuse L1	RGB	0.89	0.79	0.038	0.95	0.89	0.048
	LiDAR	0.87	0.76	0.129	0.82	0.67	0.068

**Table 5:** Correlation results from other published studies addressing grapevine geometric parameters from point cloud data. TLS: terrestrial laser scanner; MTLS: mobile terrestrial laser scanner; UAV: unmanned aerial vehicle; *H*_90_: 90th percentile of height; LAI: leaf area index; *r*: correlation coefficient; *R*^2^: coefficient of determination; RMSE: root mean square error. Black dots (•) indicate the type of sensor used in each study, namely: TLS, MTLS, and UAV.

Study	Sensors Used			Parameter	Reference Measurement	Results
	TLS	MTLS	UAV			
Escolà et al. [67]		•	•	Height	MTLS	*r*: 0.79; *R*^2^: 0.62
				*H*_90_		*r*: 0.83; *R*^2^: 0.69
Llorens et al. [50]		•		Height	Field measurements	*R*^2^: 0.23
				Volume	Ultrasonic sensor	*R*^2^: 0.56
					LAI	*R*^2^: 0.22
Chakraborty et al. [90]		•		Height	Field measurements	*r*: 0.59
				Volume	UAV canopy surface area	*r*: 0.82 convex hull *r*: 0.75 voxel grid
Rinaldi et al. [48]	•			Height	Field measurements	*R*^2^: 0.98
Torres-Sánchez et al. [31]		•	•	Height	MTLS	*R* ^2^ *: 0.76*
				Area		*R*^2^: 0.78
				Volume		*R*^2^: 0.73 (convex hull) *R*^2^: 0.85 (2.5 D volume)
Pagliai et al. [34]		•	•	Height	MTLS	*R*^2^: 0.80; RMSE: 0.124 m
				Volume		*R*^2^: 0.78; RMSE: 0.057 m^3^
Buunk et al. [91]			•	Volume	UAV LiDAR	*R*^2^: 0.70
Petrović et al. [44]		•	•	Volume	MTLS and UAV	*R*^2^: 0.92
Caruso et al. [52]			•	Height	Field measurements	*R*^2^: 0.61
				Volume		*R*^2^: 0.75

## Data Availability

Data will be made available upon reasonable request.
